# P-2046. Integrating Immunization Information System (IIS) and commercial health insurance claims data to improve COVID-19 vaccination capture among pregnant people

**DOI:** 10.1093/ofid/ofae631.2202

**Published:** 2025-01-29

**Authors:** Alexandra Stone, Wafa Tarazi, Nwanneamaka Ume, Andrea Steffens, Katherine Andrade, Garrett Gremel, Ami R Buikema, Lakshmi Panagiotakopoulos, Ruth Link-Gelles, Amadea Britton, Wenya Grace Yang

**Affiliations:** OSC, Washington, District of Columbia; Optum Serve, La Crosse, Wisconsin; Optum, La Crosse, Wisconsin; OPTUM, Eden Prairie, Minnesota; Optum, La Crosse, Wisconsin; Optum, La Crosse, Wisconsin; Optum, La Crosse, Wisconsin; Centers for Disease Control and Prevention, Atlanta, Georgia; Centers for Disease Control and Prevention, Atlanta, Georgia; Centers for Disease Control and Prevention , Atlanta, GA; Optum, La Crosse, Wisconsin

## Abstract

**Background:**

Several COVID-19 vaccine safety and effectiveness studies have used real-world data (RWD), including health insurance claims to delineate people’s vaccination history. However, COVID-19 vaccine administration is not completely captured in claims because vaccines administered in some settings, such as mass vaccination centers, do not generate insurance claims. Failure to capture complete vaccination history in claims data may result in misclassification of vaccination status leading to biased effectiveness and safety estimates. As part of a study to evaluate COVID-19 vaccine effectiveness in pregnant persons, we examined how linking claims with Immunization Information System (IIS) records improves vaccination record capture for pregnant people.

**Figure 1:**
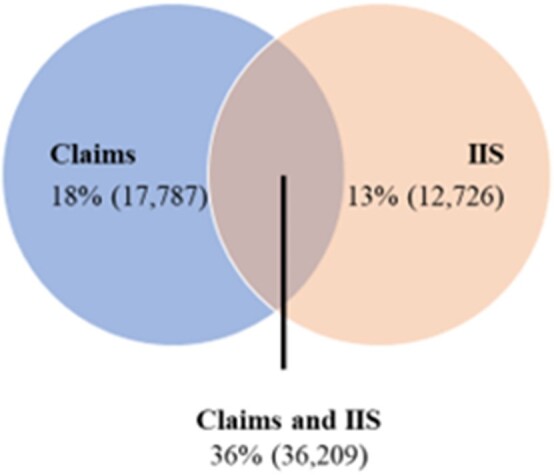
Vaccination capture in claims and IIS

**Methods:**

We identified individuals in a large commercial claims database who were pregnant between 12/11/2020 and 7/31/2023 and resided in 21 jurisdictions for which we had permission to use IIS data for public health purposes. We linked COVID-19 vaccination records from IIS and insurance claims and calculated the percentage of records that were captured in IIS only, claims only, and in both. We also compared COVID-19 vaccination capture in claims and IIS over time to assess how the completeness of IIS and claims data have changed over time.

**Results:**

This study included 101,355 pregnant people, of which 13% (12,726) had ≥1 COVID-19 vaccination record only in IIS, 18% (17,787) had ≥1 vaccination record only in the claims data, and 36% (36,209) had vaccination records in both (Figure 1). Approximately 34% (34,633) did not have a vaccination record in IIS or claims. Over time, the percentages of individuals with vaccination records in IIS, claims, and both changed; the temporal patterns aligned with a shift from vaccinations at mass vaccination centers to pharmacies and clinics.

**Conclusion:**

This study demonstrates the importance of integrating data from multiple RWD sources to capture vaccine history for vaccine effectiveness studies in pregnant people and the general population. Relying on only one data source may result in substantial misclassification of vaccinated individuals as unvaccinated, leading to biased results.

**Disclosures:**

Alexandra Stone, PhD, United Health Group: Stocks/Bonds (Public Company) Wafa Tarazi, PhD, MHPA, UnitedHealth Group: I am an employee at UHG Nwanneamaka Ume, MPH, Optum: I am an Employee of Optum, a UnitedHealth Group subsidiary Andrea Steffens, MPH, OPTUM: Employee|UnitedHealth Group: Stocks/Bonds (Public Company) Katherine Andrade, MPH, Optum: Employee|United Health Group: Stocks/Bonds (Public Company) Garrett Gremel, MS, Optum: Employee Ami R. Buikema, MPH, Optum: Employee|UnitedHealth Group: Stocks/Bonds (Public Company) Wenya Grace Yang, MPA, MA, UnitedHealth Group: I am an employee of UHG.|UnitedHealth Group: Stocks/Bonds (Public Company)

